# 2,5-Bis[2-({bis­[3-(dimethyl­aza­nium­yl)prop­yl]aza­nium­yl}meth­yl)phen­yl]-1,3,4-oxadiazole hexa­kis­(perchlorate) sesquihydrate

**DOI:** 10.1107/S1600536812047484

**Published:** 2012-11-24

**Authors:** Vieri Fusi, Mauro Formica, Eleonora Macedi, Paola Paoli, Patrizia Rossi

**Affiliations:** a Department of Basic Sciences and Fundamentals, University of Urbino, I-61029 Urbino, Italy; b Dipartimento Energetica "Sergio Stecco", University of Firenze, Via S. Marta 3, I-50139 Firenze, Italy

## Abstract

In the title hydrated salt, C_36_H_66_N_8_O^6+^·6ClO_4_^−^·1.5H_2_O, the asymmetric unit consists of a hexa­protonated [H_6_ *L*]^6+^ cation, five perchlorate anions in general positions, two on twofold rotation axes (one of which is disordered), and two water mol­ecules of crystallization in general positions, one of them disordered around a twofold crystallographic axis. In the [H_6_ *L*]^6+^ cation, two strong intra­molecular N—H⋯N hydrogen bonds occur, involving the N atoms of the oxadiazole ring as acceptors and the closest NH^+^ groups of each dipropyl­enetriamine unit. In the crystal, the [H_6_ *L*]^6+^ cations form channels along the *a-*axis direction, in which the perchlorate counter-ions and the water mol­ecules are lodged. The crystal packing features a network of N—H⋯O and O—H⋯O hydrogen bonds involving the NH^+^ groups of the [H_6_ *L*]^6+^ cation, the perchlorate anions and the water mol­ecules.

## Related literature

For 2,5 bis­[2 (chloro­meth­yl)phen­yl][1,3,4]oxadiazole, see: Formica *et al.* (2012 ▶); Wang *et al.* (1998 ▶). For systems able to recognise and signal metal cations and anions, see: Ambrosi *et al.* (2006 ▶, 2011 ▶); Ambrosi, Formica, Fusi, Giorgi, Macedi, Micheloni, Paoli *et al.* (2010 ▶)**;** Ambrosi, Formica, Fusi, Giorgi, Macedi, Micheloni, Piersanti *et al.* (2010 ▶); Bencini *et al.* (1994 ▶); Formica *et al.* (2008 ▶); Terenzi *et al.* (2012 ▶).
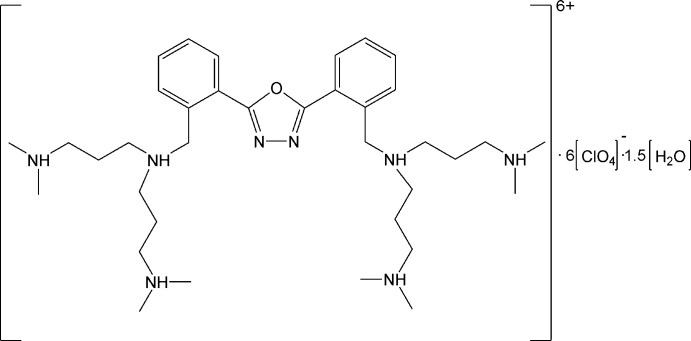


## Experimental

### 

#### Crystal data

C_36_H_66_N_8_O^6+^·6ClO_4_^−^·1.5H_2_O
*M* *_r_* = 1250.69
Monoclinic, 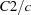

*a* = 19.5601 (7) Å
*b* = 25.0825 (8) Å
*c* = 24.2277 (9) Å
β = 113.695 (5)°
*V* = 10884.4 (7) Å^3^
*Z* = 8
Cu *K*α radiation
μ = 3.69 mm^−1^
*T* = 150 K
0.10 × 0.08 × 0.03 mm

#### Data collection

Oxford XcaliburPX diffractometer
Absorption correction: multi-scan (*CrysAlis PRO*; Oxford Diffraction, 2010 ▶) *T*_min_ = 0.728, *T*_max_ = 0.895
30246 measured reflections
10465 independent reflections
6531 reflections with *I* > 2σ(*I*)
*R*_int_ = 0.056

#### Refinement

*R*[*F* ^2^ > 2σ(*F* ^2^)] = 0.070
*wR*(*F* ^2^) = 0.219
*S* = 1.06
10465 reflections
692 parameters
3 restraints
H atoms treated by a mixture of independent and constrained refinement
Δρ_max_ = 1.48 e Å^−3^
Δρ_min_ = −0.86 e Å^−3^

### 

Data collection: *CrysAlis PRO* (Oxford Diffraction, 2010 ▶); cell refinement: *CrysAlis PRO*; data reduction: *CrysAlis PRO*; program(s) used to solve structure: *SIR97* (Altomare *et al.*, 1999 ▶); program(s) used to refine structure: *SHELXL97* (Sheldrick, 2008 ▶); molecular graphics: *ORTEP-3* (Farrugia, 2012 ▶) and *Mercury* (Macrae *et al.*, 2006 ▶); software used to prepare material for publication: *PARST97* (Nardelli, 1995 ▶).

## Supplementary Material

Crystal structure: contains datablock(s) I, global. DOI: 10.1107/S1600536812047484/lr2084sup1.cif

Structure factors: contains datablock(s) I. DOI: 10.1107/S1600536812047484/lr2084Isup2.hkl

Supplementary material file. DOI: 10.1107/S1600536812047484/lr2084Isup3.cml

Additional supplementary materials:  crystallographic information; 3D view; checkCIF report

## Figures and Tables

**Table 1:** Hydrogen-bond geometry (Å, °)

*D*—H⋯*A*	*D*—H	H⋯*A*	*D*⋯*A*	*D*—H⋯*A*
N3—H3⋯N2	0.93	1.90	2.714 (5)	145
N6—H6⋯N1	0.93	2.04	2.779 (6)	136
N5—H5⋯O1*W*	0.93	1.85	2.747 (6)	160
N4—H4⋯O2*W*	0.93	1.95	2.83 (1)	156
N4—H4⋯O2*W* ^i^	0.93	2.30	2.97 (1)	129
O1*W*—H1*WA*⋯O23^ii^	0.84 (3)	2.05 (3)	2.852 (7)	160 (3)
O1*W*—H1*WB*⋯O21^iii^	0.83 (3)	2.06 (3)	2.876 (5)	169 (3)
N7—H7⋯O22	0.93	2.26	3.000 (6)	137
N7—H7⋯O73	0.93	2.28	2.94 (1)	127
N8—H8⋯O42	0.93	2.27	3.09 (1)	146
N8—H8⋯O41^iv^	0.93	2.36	3.10 (1)	137

Symmetry codes: (i) 
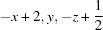
; (ii) 
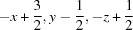
; (iii) 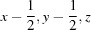; (iv) 
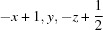
.

**Fig. 1.**
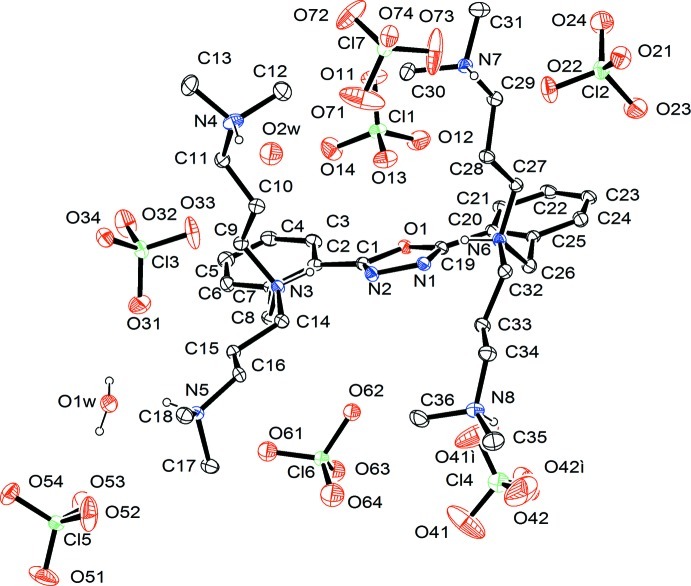
Crystal structure of the title compound with labelling and displacement ellipsoids drawn at the 20% probability level. Only one orientation for the disordered perchlorate anion (Cl6 is the chlorine atom) and the water molecule (O2W is the oxygen atom) is shown. Symmetry code: i)-x + 1, y, -z + 1/2.

**Fig. 2.**
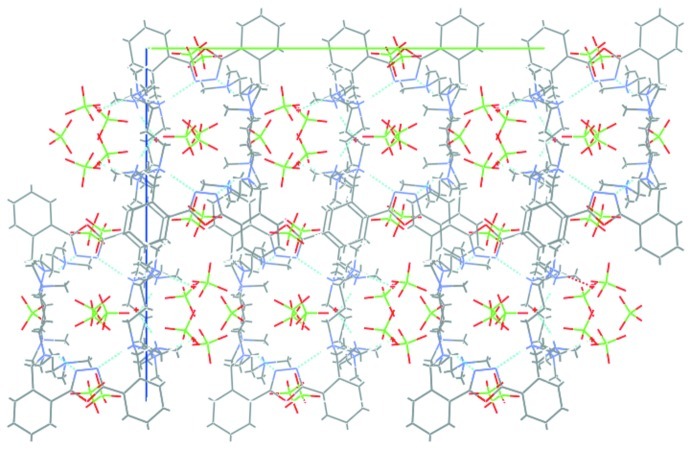
Crystal structure of the title compound with view along the a axis. Only one orientation for the disordered perchlorate anion (Cl6 is the chlorine atom) and the water molecule (O2W is the oxygen atom) is shown. Intramolecular NH+···NH bonds are depicted in turquoise.

**Fig. 3.**
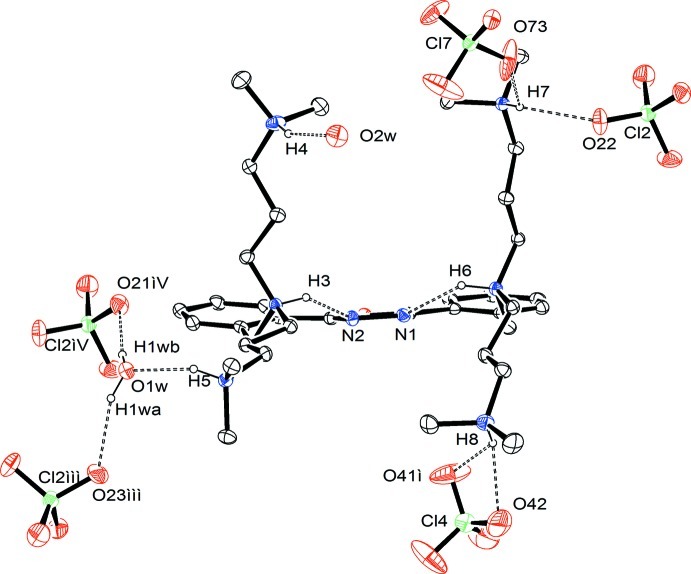
Crystal structure of the title compound showing the H-bond network (dashed lines) involving the NH+ groupings, the water molecules and the perchlorate anions. Only one orientation for the disordered water molecule (O2W is the oxygen atom) is shown. Symmetry codes: i) -x + 1; y, -z + 1/2; iii)-x + 3/2,y - 1/2,-z + 1/2; iv)x - 1/2, y - 1/2, z.

## supplementary crystallographic information

### Comment

2,5-diphenyl[1,3,4]oxadiazole (PPD) is a well known fluorophore belonging to the class of the 1,3,4-oxadiazole derivatives. It has a high photoluminescence quantum yield and both thermal and chemical stabilities (Formica *et al.*, 2012). Following our interest in systems able to recognize and signal metal cations and anions (Ambrosi *et al.* 2006; Ambrosi *et al.*, 2011; Bencini *et al.*, 1994; Formica *et al.*, 2008) we developed a class of macrocyclic polyamines incorporating PPD in their macrocyclic skeleton able to sense Zn(II) at physiological pH 7.4 as well as to intercalate DNA (Ambrosi, Formica, Fusi, Giorgi, Macedi, Micheloni, Paoli *et al.*, 2010; Ambrosi, Formica, Fusi, Giorgi, Macedi, Micheloni, Piersanti *et al.*, 2010; Terenzi *et al.*, 2012). Here we report the crystal structure of the hexaprotonated species of a new ligand *L* (2,5-bis[2-(*N*,*N*-bis(3-dimethylaminopropyl)aminomethyl)phenyl][1,3,4]oxadiazole) in which two dipropylenetriamine subunits are linked to PPD in an open-chain molecular framework. In the crystal, the are [H_6_*L*]^6+^ cations formed by the N–protonated ligand *L*, perchlorate anions and water molecules in 1:6:1.5 ratios. In particular, two perchlorate anions (Cl4 and Cl6 are the chlorine atoms) lye on binary crystallographic axes and one of them is disordered in two rotational orientations around a chlorine–oxygen bond (Cl6—O61). As a consequence their population parameters were fixed to 0.5. The same popoulation parameter was assigned to a crystallization water molecule (O2W is the oxygen atom), which is disordered around a twofold crystallographic axis. As for the [H_6_*L*]^6+^ cation, the oxadiazole mean plane slightly deviates from the mean plane defined by the phenyl rings (10.5 (2)°, Fig. 1) and, as expected, the propyl chains show all-*trans* conformations (Fig. 1). Two strong intramolecular H-bonds involve the oxadiazole nitrogen atoms N1 and N2 and the closest NH^+^ groups N6H6^+^and N3H3^+^, respectively (Table 1). In the crystal, the hexaprotonated cations form channels along the *a* axis that host the perchlorate counterions and the crystallization water molecules (Fig. 2). The latter are H-bonded to the terminal N4H4^+^ and N5H5^+^ groups of the cation (Table 1). Finally, the oxygen atoms of some perchlorate anions also are involved in a net of H-bond interactions with several NH^+^ groups of the [H_6_*L*]^6+^ cation and the water molecule (O1W is the oxygen atom). (Table 1, Fig. 3).

### Experimental

2,5 bis[2 (chloromethyl)phenyl][1,3,4]oxadiazole was prepared accordingly to literature (Wang *et al.*, 1998). 2,5-bis[2-(*N*,*N*-bis(3-dimethylaminopropyl)aminomethyl)phenyl][1,3,4]oxadiazole (*L*): over a period of 2 h, a solution of *N*,*N*-bis(3-dimethylaminopropyl)amine (1.9 g, 10 mmol) in 100 cm^3^ of anhydrous THF was added to a suspension of 2,5[bis[2-(chloromethyl)phenyl][1,3,4]oxadiazole (1.6 g, 5 mmol) and triethylamine (2.5 g, 25 mmol) in 100 cm^3^ of refluxing anhydrous THF, under nitrogen. The reaction mixture was maintained at reflux for further 12 h. Subsequently, the mixture was cooled to room temperature and then the solvent removed under reduced pressure. The residue was dissolved in CHCl_3_ (50 cm^3^) and the insoluble part filtered off; the organic layer was concentrated and the product purified by chromatography on neutral alumina eluting with CHCl_3_:MeOH (10:0.1 *v*/*v*) obtaining *L* as a yellowish oil (2.6 g, 84%). Synthesis of *L*^.^6HClO_4_^.^1.5(H_2_O): the hexa-hydroperchlorate salt was obtained in quantitative yield by adding 70% HClO_4_ to an ethanolic solution containing *L*. Crystals of *L*^.^6HClO_4_^.^1.5(H_2_O) suitable for X-ray analysis were obtained by slow evaporation of a diluted aqueous solution containing *L*^.^6HClO_4_.

### Refinement

The H atoms of the water oxygen atom O1W were found in the Fourier map and included in the refinement with their positions restrained by using the *DFIX* and DANG instructions. The constraint *U*(*H*)=1.2*U*_eq_(O) was used. For the water molecule whose oxygen atom is O2W, which is disordered around a twofold axis, a population parameter of 0.5 was used and its H atoms were not introduced in the refinement. The H atoms of [H_6_*L*]^6+^ were introduced in geometrically generated positions, riding, and the constraint *U(H)* = 1.2*U*_eq_(C,*N*) (1.5 for methyl H atoms) was applied. Both the Cl6 and the O61 of a perchlorate unit lye on a twofold axis, as a consequence the other oxygen atoms of the anion (O62, O63 and O64) are disordered with fixed popoulation parameters of 0.5. The oxygen atoms bound to Cl6 were refined with isotropic temperature factors.

### Crystal data

**Table:**

C_36_H_66_N_8_O^6+^·6ClO_4_^−^·1.5H_2_O	*F*(000) = 5240
*M**_r_* = 1250.69	*D*_x_ = 1.526 Mg m^−^^3^
Monoclinic, *C*2/*c*	Cu *K*α radiation, λ = 1.54184 Å
Hall symbol: -C 2yc	Cell parameters from 7461 reflections
*a* = 19.5601 (7) Å	θ = 4.1–72.6°
*b* = 25.0825 (8) Å	µ = 3.69 mm^−^^1^
*c* = 24.2277 (9) Å	*T* = 150 K
β = 113.695 (5)°	Prismatic, colourless
*V* = 10884.4 (7) Å^3^	0.10 × 0.08 × 0.03 mm
*Z* = 8	

### Data collection

**Table:**

Oxford XcaliburPX diffractometer	10465 independent reflections
Radiation source: Enhance (Cu) X-ray Source	6531 reflections with *I* > 2σ(*I*)
Graphite monochromator	*R*_int_ = 0.056
Detector resolution: 8.1241 pixels mm^-1^	θ_max_ = 72.7°, θ_min_ = 4.1°
ω scans	*h* = −23→22
Absorption correction: multi-scan (*CrysAlis PRO*; Oxford Diffraction, 2010)	*k* = −30→28
*T*_min_ = 0.728, *T*_max_ = 0.895	*l* = −29→22
30246 measured reflections	

### Refinement

**Table:**

Refinement on *F*^2^	Primary atom site location: structure-invariant direct methods
Least-squares matrix: full	Secondary atom site location: difference Fourier map
*R*[*F*^2^ > 2σ(*F*^2^)] = 0.070	Hydrogen site location: inferred from neighbouring sites
*wR*(*F*^2^) = 0.219	H atoms treated by a mixture of independent and constrained refinement
*S* = 1.06	*w* = 1/[σ^2^(*F*_o_^2^) + (0.1066*P*)^2^ + 23.0322*P*] where *P* = (*F*_o_^2^ + 2*F*_c_^2^)/3
10465 reflections	(Δ/σ)_max_ < 0.001
692 parameters	Δρ_max_ = 1.48 e Å^−^^3^
3 restraints	Δρ_min_ = −0.86 e Å^−^^3^

### Special details

**Table:**

Geometry. All s.u.'s (except the s.u. in the dihedral angle between two l.s. planes) are estimated using the full covariance matrix. The cell s.u.'s are taken into account individually in the estimation of s.u.'s in distances, angles and torsion angles; correlations between s.u.'s in cell parameters are only used when they are defined by crystal symmetry. An approximate (isotropic) treatment of cell s.u.'s is used for estimating s.u.'s involving l.s. planes.
Refinement. Refinement of *F*^2^ against ALL reflections. The weighted *R*-factor *wR* and goodness of fit *S* are based on *F*^2^, conventional *R*-factors *R* are based on *F*, with *F* set to zero for negative *F*^2^. The threshold expression of *F*^2^ > 2σ(*F*^2^) is used only for calculating *R*-factors(gt) *etc*. and is not relevant to the choice of reflections for refinement. *R*-factors based on *F*^2^ are statistically about twice as large as those based on *F*, and *R*- factors based on ALL data will be even larger.

### Fractional atomic coordinates and isotropic or equivalent isotropic displacement parameters (Å^2^)

**Table:**

	*x*	*y*	*z*	*U*_iso_*/*U*_eq_	Occ. (<1)
O1	0.79278 (16)	0.14810 (13)	0.50045 (13)	0.0335 (7)	
N1	0.7899 (2)	0.17588 (15)	0.41311 (16)	0.0332 (8)	
N2	0.7645 (2)	0.12311 (16)	0.40659 (16)	0.0345 (8)	
N3	0.7397 (2)	0.03182 (15)	0.34130 (16)	0.0332 (8)	
H3	0.7656	0.0624	0.3599	0.040*	
N4	0.9862 (2)	−0.0271 (2)	0.34900 (19)	0.0498 (11)	
H4	0.9682	−0.0158	0.3091	0.060*	
N5	0.5784 (2)	−0.02129 (16)	0.12725 (16)	0.0337 (8)	
H5	0.5848	−0.0548	0.1451	0.040*	
N6	0.85293 (19)	0.25580 (15)	0.36920 (15)	0.0321 (8)	
H6	0.8450	0.2197	0.3731	0.038*	
N7	1.1101 (2)	0.20308 (16)	0.39186 (17)	0.0356 (9)	
H7	1.1038	0.2162	0.3542	0.043*	
N8	0.6293 (2)	0.26003 (19)	0.17438 (19)	0.0502 (11)	
H8	0.6090	0.2784	0.1976	0.060*	
C1	0.7668 (2)	0.10832 (19)	0.4587 (2)	0.0336 (10)	
C2	0.7505 (2)	0.0563 (2)	0.4773 (2)	0.0356 (10)	
C3	0.7733 (3)	0.0468 (2)	0.5391 (2)	0.0400 (11)	
H3A	0.7957	0.0747	0.5670	0.048*	
C4	0.7636 (3)	−0.0024 (2)	0.5597 (2)	0.0434 (12)	
H4A	0.7800	−0.0085	0.6018	0.052*	
C5	0.7299 (3)	−0.0430 (2)	0.5193 (2)	0.0472 (12)	
H5A	0.7232	−0.0771	0.5334	0.057*	
C6	0.7061 (3)	−0.0335 (2)	0.4580 (2)	0.0428 (12)	
H6A	0.6831	−0.0617	0.4307	0.051*	
C7	0.7147 (2)	0.0154 (2)	0.4350 (2)	0.0362 (10)	
C8	0.6825 (2)	0.0227 (2)	0.3679 (2)	0.0382 (11)	
H8A	0.6480	0.0536	0.3575	0.046*	
H8B	0.6528	−0.0093	0.3488	0.046*	
C9	0.7970 (2)	−0.0116 (2)	0.3574 (2)	0.0378 (11)	
H9A	0.7736	−0.0446	0.3355	0.045*	
H9B	0.8157	−0.0191	0.4011	0.045*	
C10	0.8619 (3)	0.0038 (2)	0.3417 (2)	0.0379 (11)	
H10A	0.8449	0.0066	0.2974	0.045*	
H10B	0.8819	0.0390	0.3596	0.045*	
C11	0.9218 (3)	−0.0377 (2)	0.3657 (2)	0.0391 (11)	
H11A	0.9399	−0.0389	0.4102	0.047*	
H11B	0.9002	−0.0730	0.3498	0.047*	
C12	1.0350 (3)	0.0172 (3)	0.3898 (3)	0.0658 (17)	
H12A	1.0046	0.0492	0.3857	0.099*	
H12B	1.0761	0.0254	0.3780	0.099*	
H12C	1.0552	0.0052	0.4319	0.099*	
C13	1.0323 (4)	−0.0754 (3)	0.3559 (4)	0.0716 (19)	
H13A	1.0739	−0.0672	0.3446	0.107*	
H13B	1.0016	−0.1037	0.3297	0.107*	
H13C	1.0519	−0.0873	0.3979	0.107*	
C14	0.7033 (2)	0.04488 (19)	0.27488 (19)	0.0349 (10)	
H14A	0.7428	0.0529	0.2605	0.042*	
H14B	0.6728	0.0775	0.2696	0.042*	
C15	0.6538 (2)	0.00103 (19)	0.2357 (2)	0.0352 (10)	
H15A	0.6070	−0.0015	0.2420	0.042*	
H15B	0.6799	−0.0337	0.2460	0.042*	
C16	0.6368 (3)	0.01537 (19)	0.1703 (2)	0.0369 (10)	
H16A	0.6189	0.0526	0.1626	0.044*	
H16B	0.6832	0.0128	0.1632	0.044*	
C17	0.5015 (3)	−0.0028 (2)	0.1154 (2)	0.0443 (12)	
H17A	0.4960	0.0008	0.1537	0.067*	
H17B	0.4652	−0.0288	0.0898	0.067*	
H17C	0.4926	0.0318	0.0949	0.067*	
C18	0.5882 (3)	−0.0270 (2)	0.0694 (2)	0.0437 (12)	
H18A	0.6391	−0.0391	0.0779	0.066*	
H18B	0.5796	0.0075	0.0488	0.066*	
H18C	0.5523	−0.0531	0.0436	0.066*	
C19	0.8059 (2)	0.18881 (18)	0.46899 (19)	0.0313 (10)	
C20	0.8367 (2)	0.23840 (19)	0.4998 (2)	0.0333 (10)	
C21	0.8611 (2)	0.2408 (2)	0.5631 (2)	0.0381 (11)	
H21	0.8579	0.2101	0.5849	0.046*	
C22	0.8898 (3)	0.2879 (2)	0.5932 (2)	0.0425 (12)	
H22	0.9054	0.2896	0.6357	0.051*	
C23	0.8960 (3)	0.3325 (2)	0.5619 (2)	0.0418 (12)	
H23	0.9156	0.3648	0.5827	0.050*	
C24	0.8732 (3)	0.3296 (2)	0.5002 (2)	0.0402 (11)	
H24	0.8788	0.3601	0.4792	0.048*	
C25	0.8426 (2)	0.28395 (19)	0.4679 (2)	0.0327 (10)	
C26	0.8118 (3)	0.2870 (2)	0.4003 (2)	0.0367 (10)	
H26A	0.8108	0.3250	0.3888	0.044*	
H26B	0.7595	0.2743	0.3843	0.044*	
C27	0.9356 (2)	0.26593 (19)	0.39840 (19)	0.0328 (10)	
H27A	0.9541	0.2559	0.4415	0.039*	
H27B	0.9447	0.3045	0.3963	0.039*	
C28	0.9796 (2)	0.2354 (2)	0.36957 (19)	0.0349 (10)	
H28A	0.9744	0.2529	0.3314	0.042*	
H28B	0.9605	0.1985	0.3604	0.042*	
C29	1.0611 (3)	0.2350 (2)	0.4138 (2)	0.0374 (11)	
H29A	1.0797	0.2722	0.4212	0.045*	
H29B	1.0647	0.2201	0.4527	0.045*	
C30	1.0909 (3)	0.1453 (2)	0.3851 (2)	0.0449 (12)	
H30A	1.0381	0.1410	0.3582	0.067*	
H30B	1.0998	0.1301	0.4246	0.067*	
H30C	1.1220	0.1269	0.3679	0.067*	
C31	1.1903 (3)	0.2099 (3)	0.4332 (3)	0.0569 (15)	
H31A	1.2028	0.2479	0.4377	0.085*	
H31B	1.2218	0.1915	0.4163	0.085*	
H31C	1.1990	0.1947	0.4727	0.085*	
C32	0.8204 (2)	0.26915 (19)	0.30314 (19)	0.0330 (10)	
H32A	0.8493	0.2506	0.2835	0.040*	
H32B	0.8249	0.3080	0.2981	0.040*	
C33	0.7386 (3)	0.2530 (2)	0.2723 (2)	0.0386 (11)	
H33A	0.7330	0.2144	0.2782	0.046*	
H33B	0.7084	0.2731	0.2897	0.046*	
C34	0.7122 (3)	0.2653 (2)	0.2056 (2)	0.0441 (12)	
H34A	0.7271	0.3021	0.2005	0.053*	
H34B	0.7364	0.2405	0.1872	0.053*	
C35	0.6006 (4)	0.2856 (3)	0.1148 (3)	0.0702 (19)	
H35A	0.6184	0.3225	0.1189	0.105*	
H35B	0.5459	0.2854	0.0977	0.105*	
H35C	0.6182	0.2660	0.0881	0.105*	
C36	0.6051 (3)	0.2038 (3)	0.1718 (2)	0.0585 (16)	
H36A	0.6255	0.1886	0.2125	0.088*	
H36B	0.6233	0.1834	0.1460	0.088*	
H36C	0.5504	0.2022	0.1551	0.088*	
O1W	0.5627 (2)	−0.11798 (16)	0.17412 (17)	0.0506 (9)	
H1WA	0.5186 (13)	−0.125 (2)	0.168 (2)	0.061*	
H1WB	0.589 (2)	−0.123 (3)	0.2102 (12)	0.061*	
O2W	0.9681 (5)	0.0252 (4)	0.2408 (5)	0.082 (3)*	0.50
Cl1	0.97380 (6)	0.11096 (5)	0.48216 (5)	0.0437 (3)	
O11	1.0522 (2)	0.1008 (2)	0.50440 (19)	0.0709 (13)	
O12	0.9601 (3)	0.1515 (2)	0.5173 (2)	0.0907 (17)	
O13	0.9445 (2)	0.12648 (19)	0.42027 (17)	0.0638 (12)	
O14	0.9353 (3)	0.0636 (2)	0.4864 (2)	0.0781 (14)	
Cl2	1.14130 (7)	0.34632 (5)	0.33606 (6)	0.0476 (3)	
O21	1.1708 (3)	0.36700 (18)	0.29556 (19)	0.0667 (12)	
O22	1.1055 (3)	0.29673 (17)	0.3134 (2)	0.0767 (14)	
O23	1.0892 (3)	0.3846 (2)	0.3400 (2)	0.0851 (16)	
O24	1.2018 (3)	0.3390 (2)	0.3932 (2)	0.0857 (15)	
Cl3	0.80344 (7)	−0.09755 (5)	0.20105 (5)	0.0429 (3)	
O31	0.7242 (3)	−0.0925 (2)	0.1759 (3)	0.0855 (16)	
O32	0.8267 (3)	−0.1218 (2)	0.25926 (18)	0.0835 (16)	
O33	0.8382 (3)	−0.04650 (17)	0.2075 (2)	0.0853 (17)	
O34	0.8237 (2)	−0.13032 (15)	0.16220 (16)	0.0512 (9)	
Cl4	0.5000	0.28569 (11)	0.2500	0.0731 (6)	
O41	0.4355 (4)	0.2570 (4)	0.2281 (3)	0.165 (4)	
O42	0.5038 (5)	0.3126 (3)	0.2020 (5)	0.189 (4)	
Cl5	0.41888 (7)	−0.14088 (5)	−0.02396 (6)	0.0463 (3)	
O51	0.3437 (3)	−0.1463 (3)	−0.0591 (4)	0.180 (5)	
O52	0.4489 (4)	−0.1046 (2)	−0.0536 (3)	0.113 (2)	
O53	0.4347 (4)	−0.1202 (3)	0.0338 (2)	0.1028 (19)	
O54	0.4543 (3)	−0.19040 (17)	−0.02144 (18)	0.0673 (12)	
Cl6	0.5000	0.09905 (7)	0.2500	0.0434 (4)	
O61	0.5000	0.0423 (2)	0.2500	0.0626 (15)*	
O62	0.5722 (4)	0.1244 (3)	0.2816 (3)	0.0475 (17)*	0.50
O63	0.4696 (5)	0.1094 (4)	0.2973 (4)	0.075 (3)*	0.50
O64	0.4592 (7)	0.1181 (5)	0.1966 (5)	0.091 (3)*	0.50
Cl7	1.14682 (7)	0.13151 (5)	0.25946 (5)	0.0441 (3)	
O71	1.0715 (3)	0.1145 (4)	0.2315 (3)	0.163 (4)	
O72	1.1825 (4)	0.0908 (2)	0.2980 (3)	0.110 (2)	
O73	1.1453 (5)	0.17912 (19)	0.2873 (3)	0.143 (3)	
O74	1.1737 (2)	0.13856 (15)	0.21346 (16)	0.0516 (9)	

### Atomic displacement parameters (Å^2^)

**Table:**

	*U*^11^	*U*^22^	*U*^33^	*U*^12^	*U*^13^	*U*^23^
O1	0.0320 (16)	0.0381 (18)	0.0291 (15)	−0.0032 (13)	0.0110 (12)	−0.0035 (13)
N1	0.0297 (18)	0.035 (2)	0.0334 (19)	−0.0025 (16)	0.0106 (15)	−0.0022 (16)
N2	0.037 (2)	0.034 (2)	0.0306 (18)	−0.0044 (17)	0.0115 (15)	−0.0022 (16)
N3	0.0320 (19)	0.032 (2)	0.0334 (19)	−0.0056 (16)	0.0104 (15)	−0.0039 (16)
N4	0.045 (2)	0.066 (3)	0.042 (2)	0.015 (2)	0.0214 (19)	0.008 (2)
N5	0.0316 (19)	0.035 (2)	0.0317 (19)	−0.0001 (16)	0.0094 (15)	−0.0021 (16)
N6	0.0325 (19)	0.031 (2)	0.0316 (18)	−0.0017 (16)	0.0120 (15)	−0.0049 (15)
N7	0.032 (2)	0.037 (2)	0.039 (2)	0.0019 (17)	0.0151 (16)	0.0031 (17)
N8	0.040 (2)	0.054 (3)	0.045 (2)	0.002 (2)	0.0059 (19)	0.002 (2)
C1	0.029 (2)	0.035 (3)	0.034 (2)	0.0008 (19)	0.0103 (18)	−0.0058 (19)
C2	0.024 (2)	0.040 (3)	0.041 (2)	0.0010 (19)	0.0108 (18)	0.000 (2)
C3	0.032 (2)	0.049 (3)	0.038 (2)	−0.005 (2)	0.0141 (19)	0.001 (2)
C4	0.036 (3)	0.055 (3)	0.040 (3)	−0.002 (2)	0.017 (2)	0.007 (2)
C5	0.040 (3)	0.047 (3)	0.060 (3)	−0.002 (2)	0.026 (2)	0.009 (3)
C6	0.039 (3)	0.037 (3)	0.054 (3)	−0.005 (2)	0.020 (2)	−0.005 (2)
C7	0.026 (2)	0.044 (3)	0.037 (2)	−0.003 (2)	0.0104 (18)	−0.004 (2)
C8	0.030 (2)	0.044 (3)	0.042 (3)	−0.004 (2)	0.0154 (19)	−0.005 (2)
C9	0.034 (2)	0.036 (3)	0.037 (2)	0.002 (2)	0.0080 (19)	−0.001 (2)
C10	0.036 (2)	0.044 (3)	0.035 (2)	−0.001 (2)	0.0150 (19)	−0.001 (2)
C11	0.038 (2)	0.038 (3)	0.043 (3)	−0.001 (2)	0.017 (2)	0.001 (2)
C12	0.054 (3)	0.057 (4)	0.089 (5)	−0.008 (3)	0.030 (3)	−0.008 (3)
C13	0.068 (4)	0.049 (4)	0.106 (5)	0.011 (3)	0.043 (4)	−0.019 (4)
C14	0.032 (2)	0.035 (3)	0.034 (2)	−0.003 (2)	0.0097 (18)	−0.0017 (19)
C15	0.032 (2)	0.032 (2)	0.039 (2)	−0.0014 (19)	0.0115 (19)	−0.005 (2)
C16	0.036 (2)	0.030 (2)	0.039 (2)	−0.007 (2)	0.0094 (19)	−0.007 (2)
C17	0.032 (2)	0.057 (3)	0.041 (3)	−0.002 (2)	0.012 (2)	−0.012 (2)
C18	0.043 (3)	0.050 (3)	0.038 (3)	−0.003 (2)	0.016 (2)	−0.005 (2)
C19	0.027 (2)	0.034 (3)	0.034 (2)	−0.0027 (18)	0.0125 (17)	−0.0071 (19)
C20	0.025 (2)	0.036 (3)	0.038 (2)	0.0005 (18)	0.0114 (18)	−0.010 (2)
C21	0.032 (2)	0.047 (3)	0.032 (2)	−0.001 (2)	0.0091 (18)	−0.007 (2)
C22	0.032 (2)	0.057 (3)	0.034 (2)	−0.001 (2)	0.0087 (19)	−0.017 (2)
C23	0.030 (2)	0.044 (3)	0.047 (3)	−0.001 (2)	0.011 (2)	−0.017 (2)
C24	0.038 (3)	0.040 (3)	0.046 (3)	−0.002 (2)	0.021 (2)	−0.005 (2)
C25	0.026 (2)	0.034 (3)	0.037 (2)	0.0032 (19)	0.0117 (18)	−0.0041 (19)
C26	0.036 (2)	0.037 (3)	0.039 (2)	0.000 (2)	0.017 (2)	−0.007 (2)
C27	0.032 (2)	0.033 (2)	0.031 (2)	−0.0028 (19)	0.0102 (18)	−0.0024 (18)
C28	0.031 (2)	0.042 (3)	0.029 (2)	−0.002 (2)	0.0085 (17)	−0.0052 (19)
C29	0.036 (2)	0.042 (3)	0.036 (2)	−0.001 (2)	0.0152 (19)	−0.004 (2)
C30	0.050 (3)	0.036 (3)	0.054 (3)	0.006 (2)	0.027 (2)	0.004 (2)
C31	0.035 (3)	0.070 (4)	0.059 (3)	0.003 (3)	0.012 (2)	0.005 (3)
C32	0.036 (2)	0.033 (3)	0.030 (2)	−0.0010 (19)	0.0126 (18)	0.0032 (18)
C33	0.036 (2)	0.041 (3)	0.036 (2)	−0.004 (2)	0.0113 (19)	0.000 (2)
C34	0.038 (3)	0.052 (3)	0.040 (3)	−0.003 (2)	0.012 (2)	0.006 (2)
C35	0.068 (4)	0.072 (5)	0.045 (3)	0.007 (3)	−0.003 (3)	0.013 (3)
C36	0.049 (3)	0.063 (4)	0.045 (3)	−0.012 (3)	0.000 (2)	−0.003 (3)
O1W	0.047 (2)	0.046 (2)	0.060 (2)	−0.0051 (18)	0.0228 (18)	0.0028 (19)
Cl1	0.0394 (6)	0.0536 (8)	0.0401 (6)	0.0062 (5)	0.0179 (5)	0.0096 (5)
O11	0.044 (2)	0.107 (4)	0.062 (3)	0.021 (2)	0.0223 (19)	0.028 (2)
O12	0.074 (3)	0.096 (4)	0.076 (3)	0.025 (3)	0.004 (2)	−0.035 (3)
O13	0.056 (2)	0.089 (3)	0.048 (2)	0.013 (2)	0.0230 (18)	0.026 (2)
O14	0.085 (3)	0.074 (3)	0.071 (3)	−0.022 (3)	0.027 (2)	0.020 (2)
Cl2	0.0554 (7)	0.0435 (7)	0.0517 (7)	−0.0006 (6)	0.0295 (6)	−0.0031 (6)
O21	0.078 (3)	0.064 (3)	0.072 (3)	−0.004 (2)	0.045 (2)	0.011 (2)
O22	0.109 (4)	0.046 (3)	0.071 (3)	−0.025 (3)	0.032 (3)	−0.001 (2)
O23	0.060 (3)	0.093 (4)	0.111 (4)	0.005 (3)	0.044 (3)	−0.037 (3)
O24	0.091 (4)	0.083 (4)	0.062 (3)	−0.001 (3)	0.010 (2)	0.008 (3)
Cl3	0.0525 (7)	0.0393 (7)	0.0397 (6)	−0.0092 (5)	0.0214 (5)	−0.0057 (5)
O31	0.055 (3)	0.083 (4)	0.114 (4)	0.017 (2)	0.030 (3)	−0.023 (3)
O32	0.130 (4)	0.078 (3)	0.041 (2)	−0.028 (3)	0.032 (2)	0.000 (2)
O33	0.157 (5)	0.045 (3)	0.077 (3)	−0.048 (3)	0.071 (3)	−0.023 (2)
O34	0.053 (2)	0.055 (2)	0.054 (2)	−0.0083 (18)	0.0308 (18)	−0.0141 (18)
Cl4	0.0630 (14)	0.0767 (17)	0.0780 (15)	0.000	0.0266 (12)	0.000
O41	0.129 (6)	0.282 (11)	0.123 (5)	−0.123 (7)	0.091 (5)	−0.102 (6)
O42	0.147 (7)	0.145 (7)	0.283 (11)	0.043 (6)	0.096 (7)	0.147 (8)
Cl5	0.0354 (6)	0.0448 (7)	0.0524 (7)	−0.0001 (5)	0.0111 (5)	−0.0112 (6)
O51	0.035 (3)	0.192 (8)	0.273 (9)	−0.001 (4)	0.019 (4)	−0.169 (7)
O52	0.206 (7)	0.046 (3)	0.086 (4)	−0.015 (4)	0.058 (4)	0.011 (3)
O53	0.133 (5)	0.113 (5)	0.074 (3)	−0.015 (4)	0.054 (3)	−0.047 (3)
O54	0.087 (3)	0.051 (3)	0.058 (2)	0.023 (2)	0.024 (2)	0.013 (2)
Cl6	0.0347 (8)	0.0388 (9)	0.0529 (10)	0.000	0.0138 (7)	0.000
Cl7	0.0518 (7)	0.0391 (7)	0.0441 (6)	0.0056 (5)	0.0218 (5)	0.0014 (5)
O71	0.065 (4)	0.335 (12)	0.090 (4)	−0.041 (5)	0.032 (3)	0.011 (6)
O72	0.152 (5)	0.112 (5)	0.095 (4)	0.084 (4)	0.079 (4)	0.062 (3)
O73	0.336 (11)	0.038 (3)	0.147 (5)	−0.007 (4)	0.195 (7)	−0.008 (3)
O74	0.071 (3)	0.048 (2)	0.047 (2)	0.0028 (19)	0.0356 (18)	−0.0017 (17)

### Geometric parameters (Å, º)

**Table:**

O1—C19	1.359 (5)	C19—C20	1.451 (6)
O1—C1	1.365 (5)	C20—C25	1.410 (7)
N1—C19	1.302 (5)	C20—C21	1.411 (6)
N1—N2	1.400 (5)	C21—C22	1.383 (7)
N2—C1	1.299 (6)	C21—H21	0.9500
N3—C9	1.498 (6)	C22—C23	1.384 (8)
N3—C14	1.511 (6)	C22—H22	0.9500
N3—C8	1.517 (6)	C23—C24	1.379 (7)
N3—H3	0.9300	C23—H23	0.9500
N4—C13	1.479 (7)	C24—C25	1.380 (7)
N4—C11	1.494 (6)	C24—H24	0.9500
N4—C12	1.537 (8)	C25—C26	1.502 (6)
N4—H4	0.9300	C26—H26A	0.9900
N5—C17	1.489 (6)	C26—H26B	0.9900
N5—C18	1.495 (6)	C27—C28	1.515 (6)
N5—C16	1.510 (5)	C27—H27A	0.9900
N5—H5	0.9300	C27—H27B	0.9900
N6—C27	1.503 (5)	C28—C29	1.524 (6)
N6—C32	1.503 (5)	C28—H28A	0.9900
N6—C26	1.522 (6)	C28—H28B	0.9900
N6—H6	0.9300	C29—H29A	0.9900
N7—C30	1.490 (6)	C29—H29B	0.9900
N7—C31	1.494 (6)	C30—H30A	0.9800
N7—C29	1.501 (6)	C30—H30B	0.9800
N7—H7	0.9300	C30—H30C	0.9800
N8—C35	1.469 (7)	C31—H31A	0.9800
N8—C36	1.481 (7)	C31—H31B	0.9800
N8—C34	1.495 (6)	C31—H31C	0.9800
N8—H8	0.9300	C32—C33	1.524 (6)
C1—C2	1.458 (7)	C32—H32A	0.9900
C2—C3	1.399 (6)	C32—H32B	0.9900
C2—C7	1.421 (7)	C33—C34	1.516 (6)
C3—C4	1.372 (7)	C33—H33A	0.9900
C3—H3A	0.9500	C33—H33B	0.9900
C4—C5	1.383 (8)	C34—H34A	0.9900
C4—H4A	0.9500	C34—H34B	0.9900
C5—C6	1.387 (7)	C35—H35A	0.9800
C5—H5A	0.9500	C35—H35B	0.9800
C6—C7	1.385 (7)	C35—H35C	0.9800
C6—H6A	0.9500	C36—H36A	0.9800
C7—C8	1.499 (6)	C36—H36B	0.9800
C8—H8A	0.9900	C36—H36C	0.9800
C8—H8B	0.9900	O1W—H1WA	0.836 (19)
C9—C10	1.516 (7)	O1W—H1WB	0.828 (19)
C9—H9A	0.9900	Cl1—O12	1.419 (5)
C9—H9B	0.9900	Cl1—O13	1.427 (4)
C10—C11	1.498 (7)	Cl1—O11	1.429 (4)
C10—H10A	0.9900	Cl1—O14	1.431 (5)
C10—H10B	0.9900	Cl2—O21	1.421 (4)
C11—H11A	0.9900	Cl2—O22	1.425 (4)
C11—H11B	0.9900	Cl2—O24	1.427 (5)
C12—H12A	0.9800	Cl2—O23	1.430 (5)
C12—H12B	0.9800	Cl3—O34	1.421 (4)
C12—H12C	0.9800	Cl3—O31	1.425 (5)
C13—H13A	0.9800	Cl3—O33	1.429 (4)
C13—H13B	0.9800	Cl3—O32	1.432 (4)
C13—H13C	0.9800	Cl4—O41	1.361 (6)
C14—C15	1.519 (6)	Cl4—O42	1.371 (8)
C14—H14A	0.9900	Cl5—O51	1.378 (5)
C14—H14B	0.9900	Cl5—O53	1.406 (5)
C15—C16	1.525 (6)	Cl5—O54	1.411 (4)
C15—H15A	0.9900	Cl5—O52	1.423 (6)
C15—H15B	0.9900	Cl6—O64	1.308 (11)
C16—H16A	0.9900	Cl6—O61	1.423 (6)
C16—H16B	0.9900	Cl6—O62	1.455 (7)
C17—H17A	0.9800	Cl6—O63	1.512 (10)
C17—H17B	0.9800	Cl7—O72	1.370 (5)
C17—H17C	0.9800	Cl7—O73	1.377 (5)
C18—H18A	0.9800	Cl7—O71	1.417 (6)
C18—H18B	0.9800	Cl7—O74	1.421 (4)
C18—H18C	0.9800		
			
C19—O1—C1	103.4 (3)	O1—C19—C20	119.2 (4)
C19—N1—N2	106.2 (4)	C25—C20—C21	119.6 (4)
C1—N2—N1	106.8 (4)	C25—C20—C19	121.4 (4)
C9—N3—C14	114.8 (3)	C21—C20—C19	119.0 (4)
C9—N3—C8	112.5 (4)	C22—C21—C20	119.9 (5)
C14—N3—C8	111.8 (3)	C22—C21—H21	120.0
C9—N3—H3	105.6	C20—C21—H21	120.0
C14—N3—H3	105.6	C21—C22—C23	120.5 (4)
C8—N3—H3	105.6	C21—C22—H22	119.8
C13—N4—C11	111.5 (5)	C23—C22—H22	119.8
C13—N4—C12	109.1 (5)	C24—C23—C22	119.3 (5)
C11—N4—C12	109.1 (4)	C24—C23—H23	120.3
C13—N4—H4	109.0	C22—C23—H23	120.3
C11—N4—H4	109.0	C23—C24—C25	122.4 (5)
C12—N4—H4	109.0	C23—C24—H24	118.8
C17—N5—C18	110.4 (3)	C25—C24—H24	118.8
C17—N5—C16	111.7 (4)	C24—C25—C20	118.3 (4)
C18—N5—C16	111.1 (4)	C24—C25—C26	117.9 (4)
C17—N5—H5	107.8	C20—C25—C26	123.6 (4)
C18—N5—H5	107.8	C25—C26—N6	117.0 (4)
C16—N5—H5	107.8	C25—C26—H26A	108.1
C27—N6—C32	112.2 (3)	N6—C26—H26A	108.1
C27—N6—C26	111.2 (3)	C25—C26—H26B	108.1
C32—N6—C26	109.4 (3)	N6—C26—H26B	108.1
C27—N6—H6	107.9	H26A—C26—H26B	107.3
C32—N6—H6	107.9	N6—C27—C28	113.6 (3)
C26—N6—H6	107.9	N6—C27—H27A	108.8
C30—N7—C31	109.6 (4)	C28—C27—H27A	108.8
C30—N7—C29	112.9 (4)	N6—C27—H27B	108.8
C31—N7—C29	110.4 (4)	C28—C27—H27B	108.8
C30—N7—H7	107.9	H27A—C27—H27B	107.7
C31—N7—H7	107.9	C27—C28—C29	107.9 (4)
C29—N7—H7	107.9	C27—C28—H28A	110.1
C35—N8—C36	112.6 (5)	C29—C28—H28A	110.1
C35—N8—C34	111.6 (5)	C27—C28—H28B	110.1
C36—N8—C34	111.7 (4)	C29—C28—H28B	110.1
C35—N8—H8	106.9	H28A—C28—H28B	108.4
C36—N8—H8	106.9	N7—C29—C28	113.0 (4)
C34—N8—H8	106.9	N7—C29—H29A	109.0
N2—C1—O1	111.6 (4)	C28—C29—H29A	109.0
N2—C1—C2	129.1 (4)	N7—C29—H29B	109.0
O1—C1—C2	119.2 (4)	C28—C29—H29B	109.0
C3—C2—C7	120.2 (5)	H29A—C29—H29B	107.8
C3—C2—C1	117.9 (4)	N7—C30—H30A	109.5
C7—C2—C1	121.9 (4)	N7—C30—H30B	109.5
C4—C3—C2	120.8 (5)	H30A—C30—H30B	109.5
C4—C3—H3A	119.6	N7—C30—H30C	109.5
C2—C3—H3A	119.6	H30A—C30—H30C	109.5
C3—C4—C5	120.0 (5)	H30B—C30—H30C	109.5
C3—C4—H4A	120.0	N7—C31—H31A	109.5
C5—C4—H4A	120.0	N7—C31—H31B	109.5
C4—C5—C6	119.5 (5)	H31A—C31—H31B	109.5
C4—C5—H5A	120.2	N7—C31—H31C	109.5
C6—C5—H5A	120.2	H31A—C31—H31C	109.5
C7—C6—C5	122.7 (5)	H31B—C31—H31C	109.5
C7—C6—H6A	118.7	N6—C32—C33	112.0 (4)
C5—C6—H6A	118.7	N6—C32—H32A	109.2
C6—C7—C2	116.9 (4)	C33—C32—H32A	109.2
C6—C7—C8	118.6 (4)	N6—C32—H32B	109.2
C2—C7—C8	124.4 (5)	C33—C32—H32B	109.2
C7—C8—N3	114.8 (4)	H32A—C32—H32B	107.9
C7—C8—H8A	108.6	C34—C33—C32	108.0 (4)
N3—C8—H8A	108.6	C34—C33—H33A	110.1
C7—C8—H8B	108.6	C32—C33—H33A	110.1
N3—C8—H8B	108.6	C34—C33—H33B	110.1
H8A—C8—H8B	107.6	C32—C33—H33B	110.1
N3—C9—C10	111.3 (4)	H33A—C33—H33B	108.4
N3—C9—H9A	109.4	N8—C34—C33	110.9 (4)
C10—C9—H9A	109.4	N8—C34—H34A	109.5
N3—C9—H9B	109.4	C33—C34—H34A	109.5
C10—C9—H9B	109.4	N8—C34—H34B	109.5
H9A—C9—H9B	108.0	C33—C34—H34B	109.5
C11—C10—C9	109.1 (4)	H34A—C34—H34B	108.0
C11—C10—H10A	109.9	N8—C35—H35A	109.5
C9—C10—H10A	109.9	N8—C35—H35B	109.5
C11—C10—H10B	109.9	H35A—C35—H35B	109.5
C9—C10—H10B	109.9	N8—C35—H35C	109.5
H10A—C10—H10B	108.3	H35A—C35—H35C	109.5
N4—C11—C10	112.6 (4)	H35B—C35—H35C	109.5
N4—C11—H11A	109.1	N8—C36—H36A	109.5
C10—C11—H11A	109.1	N8—C36—H36B	109.5
N4—C11—H11B	109.1	H36A—C36—H36B	109.5
C10—C11—H11B	109.1	N8—C36—H36C	109.5
H11A—C11—H11B	107.8	H36A—C36—H36C	109.5
N4—C12—H12A	109.5	H36B—C36—H36C	109.5
N4—C12—H12B	109.5	H1WA—O1W—H1WB	108 (3)
H12A—C12—H12B	109.5	O12—Cl1—O13	109.9 (3)
N4—C12—H12C	109.5	O12—Cl1—O11	109.5 (3)
H12A—C12—H12C	109.5	O13—Cl1—O11	111.2 (2)
H12B—C12—H12C	109.5	O12—Cl1—O14	108.7 (4)
N4—C13—H13A	109.5	O13—Cl1—O14	107.5 (3)
N4—C13—H13B	109.5	O11—Cl1—O14	109.9 (3)
H13A—C13—H13B	109.5	O21—Cl2—O22	109.0 (3)
N4—C13—H13C	109.5	O21—Cl2—O24	108.0 (3)
H13A—C13—H13C	109.5	O22—Cl2—O24	110.4 (3)
H13B—C13—H13C	109.5	O21—Cl2—O23	107.4 (3)
N3—C14—C15	114.7 (4)	O22—Cl2—O23	111.0 (3)
N3—C14—H14A	108.6	O24—Cl2—O23	110.9 (3)
C15—C14—H14A	108.6	O34—Cl3—O31	108.3 (3)
N3—C14—H14B	108.6	O34—Cl3—O33	110.1 (2)
C15—C14—H14B	108.6	O31—Cl3—O33	110.8 (3)
H14A—C14—H14B	107.6	O34—Cl3—O32	110.1 (3)
C14—C15—C16	107.3 (4)	O31—Cl3—O32	108.6 (3)
C14—C15—H15A	110.3	O33—Cl3—O32	108.9 (3)
C16—C15—H15A	110.3	O41^i^—Cl4—O41	116.1 (9)
C14—C15—H15B	110.3	O41^i^—Cl4—O42	103.5 (4)
C16—C15—H15B	110.3	O41—Cl4—O42	106.7 (6)
H15A—C15—H15B	108.5	O41^i^—Cl4—O42^i^	106.7 (6)
N5—C16—C15	111.2 (4)	O41—Cl4—O42^i^	103.5 (4)
N5—C16—H16A	109.4	O42—Cl4—O42^i^	121.0 (10)
C15—C16—H16A	109.4	O51—Cl5—O53	113.9 (4)
N5—C16—H16B	109.4	O51—Cl5—O54	108.8 (4)
C15—C16—H16B	109.4	O53—Cl5—O54	111.9 (3)
H16A—C16—H16B	108.0	O51—Cl5—O52	107.8 (6)
N5—C17—H17A	109.5	O53—Cl5—O52	107.2 (4)
N5—C17—H17B	109.5	O54—Cl5—O52	106.8 (3)
H17A—C17—H17B	109.5	O64—Cl6—O61	111.4 (5)
N5—C17—H17C	109.5	O64—Cl6—O62	116.0 (5)
H17A—C17—H17C	109.5	O61—Cl6—O62	115.9 (3)
H17B—C17—H17C	109.5	O64—Cl6—O63	114.8 (6)
N5—C18—H18A	109.5	O61—Cl6—O63	99.9 (4)
N5—C18—H18B	109.5	O62—Cl6—O63	97.0 (5)
H18A—C18—H18B	109.5	O72—Cl7—O73	114.7 (4)
N5—C18—H18C	109.5	O72—Cl7—O71	104.3 (5)
H18A—C18—H18C	109.5	O73—Cl7—O71	105.9 (6)
H18B—C18—H18C	109.5	O72—Cl7—O74	112.3 (3)
N1—C19—O1	112.0 (4)	O73—Cl7—O74	111.3 (3)
N1—C19—C20	128.7 (4)	O71—Cl7—O74	107.6 (3)

Symmetry code: (i) −*x*+1, *y*, −*z*+1/2.

### Hydrogen-bond geometry (Å, º)

**Table:**

*D*—H···*A*	*D*—H	H···*A*	*D*···*A*	*D*—H···*A*
N3—H3···N2	0.93	1.90	2.714 (5)	145
N6—H6···N1	0.93	2.04	2.779 (6)	136
N5—H5···O1*W*	0.93	1.85	2.747 (6)	160
N4—H4···O2*W*	0.93	1.95	2.83 (1)	156
N4—H4···O2*W*^ii^	0.93	2.30	2.97 (1)	129
O1*W*—H1*WA*···O23^iii^	0.84 (3)	2.05 (3)	2.852 (7)	160 (3)
O1*W*—H1*WB*···O21^iv^	0.83 (3)	2.06 (3)	2.876 (5)	169 (3)
N7—H7···O22	0.93	2.26	3.000 (6)	137
N7—H7···O73	0.93	2.28	2.94 (1)	127
N8—H8···O42	0.93	2.27	3.09 (1)	146
N8—H8···O41^i^	0.93	2.36	3.10 (1)	137

Symmetry codes: (i) −*x*+1, *y*, −*z*+1/2; (ii) −*x*+2, *y*, −*z*+1/2; (iii) −*x*+3/2, *y*−1/2, −*z*+1/2; (iv) *x*−1/2, *y*−1/2, *z*.
